# A hybrid brain-muscle-machine interface for stroke rehabilitation: Usability and functionality validation in a 2-week intensive intervention

**DOI:** 10.3389/fbioe.2024.1330330

**Published:** 2024-04-12

**Authors:** Andrea Sarasola-Sanz, Andreas M. Ray, Ainhoa Insausti-Delgado, Nerea Irastorza-Landa, Wala Jaser Mahmoud, Doris Brötz, Carlos Bibián-Nogueras, Florian Helmhold, Christoph Zrenner, Ulf Ziemann, Eduardo López-Larraz, Ander Ramos-Murguialday

**Affiliations:** ^1^ Health Unit, TECNALIA, Basque Research and Technology Alliance (BRTA), San Sebastian, Spain; ^2^ Institute of Medical Psychology and Behavioral Neurobiology, University of Tübingen, Tübingen, Germany; ^3^ Department of Neurology and Stroke, University Tübingen, Tübingen, Germany; ^4^ Hertie Institute for Clinical Brain Research, University Tübingen, Tübingen, Germany; ^5^ Temerty Centre for Therapeutic Brain Intervention, Centre for Addiction and Mental Health, Toronto, Canada; ^6^ Department of Psychiatry, University of Toronto, Toronto, Canada; ^7^ Institute for Biomedical Engineering, University of Toronto, Toronto, Canada; ^8^ Bitbrain, Zaragoza, Spain

**Keywords:** hybrid brain-muscle-machine interface, stroke, upper limb rehabilitation, multidegree of freedom exoskeleton, bio-inspired motor control, cortico-muscular connection, pilot clinical trial

## Abstract

**Introduction:** The primary constraint of non-invasive brain-machine interfaces (BMIs) in stroke rehabilitation lies in the poor spatial resolution of motor intention related neural activity capture. To address this limitation, hybrid brain-muscle-machine interfaces (hBMIs) have been suggested as superior alternatives. These hybrid interfaces incorporate supplementary input data from muscle signals to enhance the accuracy, smoothness and dexterity of rehabilitation device control. Nevertheless, determining the distribution of control between the brain and muscles is a complex task, particularly when applied to exoskeletons with multiple degrees of freedom (DoFs). Here we present a feasibility, usability and functionality study of a bio-inspired hybrid brain-muscle machine interface to continuously control an upper limb exoskeleton with 7 DoFs.

**Methods:** The system implements a hierarchical control strategy that follows the biologically natural motor command pathway from the brain to the muscles. Additionally, it employs an innovative mirror myoelectric decoder, offering patients a reference model to assist them in relearning healthy muscle activation patterns during training. Furthermore, the multi-DoF exoskeleton enables the practice of coordinated arm and hand movements, which may facilitate the early use of the affected arm in daily life activities. In this pilot trial six chronic and severely paralyzed patients controlled the multi-DoF exoskeleton using their brain and muscle activity. The intervention consisted of 2 weeks of hBMI training of functional tasks with the system followed by physiotherapy. Patients’ feedback was collected during and after the trial by means of several feedback questionnaires. Assessment sessions comprised clinical scales and neurophysiological measurements, conducted prior to, immediately following the intervention, and at a 2-week follow-up.

**Results:** Patients’ feedback indicates a great adoption of the technology and their confidence in its rehabilitation potential. Half of the patients showed improvements in their arm function and 83% improved their hand function. Furthermore, we found improved patterns of muscle activation as well as increased motor evoked potentials after the intervention.

**Discussion:** This underscores the significant potential of bio-inspired interfaces that engage the entire nervous system, spanning from the brain to the muscles, for the rehabilitation of stroke patients, even those who are severely paralyzed and in the chronic phase.

## 1 Introduction

After a stroke, cortical and subcortical damage disrupts signals descending from the motor cortex to the spinal cord. The resulting recruitment patterns of muscles are pathological (Roh et al., 2013; Urra et al., 2014). Stroke is among the leading causes of disability and more than 30% of all survivors show limited recovery of their motor abilities (Young and Forster, 2007; Langhorne et al., 2009).

In a rehabilitation intervention, training of functional movements involving multiple degrees of freedom (DoFs) of the upper limb has been shown to facilitate the translation of the regained motor skills to activities of daily living (Takeuchi and Izumi, 2012; Garcia-Cossio et al., 2013). Nonetheless, effectively and proficiently controlling rehabilitation exoskeletons with multiple DoFs using neurophysiological signals remains a challenge. This is due to the dynamics and the mechanical constraints of the exoskeletons, as well as the complexity of modelling human motor control mechanisms (Sartori et al., 2018). This has been reflected in the absence of multi-DoF rehabilitation systems that can offer an accurate method to train functional tasks.

Conventional non-invasive Brain-Machine Interface (BMI) therapies have shown promising results in rehabilitation of the upper limb of stroke patients. However, the poor quality of the signal captured from the brain constitutes a major limitation. Currently, precise control of a rehabilitation robot complex enough to be able to train functional tasks is not possible with such systems.

Hybrid brain-muscle-machine interfaces (hBMI) have been proposed to overcome these limitations. These systems supplement the brain signals used for decoding the patient´s intention by including electromyography (EMG) as a control signal (Leeb et al., 2011; Müller-Putz et al., 2011; Lalitharatne et al., 2013; Kawase et al., 2017; Sarasola-Sanz et al., 2017; Loopez-Larraz et al., 2018; Zhang et al., 2019) or by using features based on the functional connection between the brain and the muscle activity, such as the corticomuscular coherence (Chowdhury et al., 2019; Colamarino et al., 2021; de Seta et al., 2022; Guo et al., 2022; Pichiorri et al., 2023). This, in turn, improves the accuracy of movement intention detection and the feedback given to stroke patients (López-Larraz et al., 2019). Moreover, these types of hBMIs establish a biologically inspired hierarchical control flow, reinforcing the brain-to-muscle connection and thus, acting both at the central and peripheral nervous systems for an integral rehabilitation.

Although the control of these hBMIs and the accuracy of feedback have advanced significantly in recent years, to our knowledge, there is no study that has achieved continuous control of an upper limb exoskeleton with multiple degrees of freedom (DoFs) for post-stroke rehabilitation. Most systems are limited to triggering a predetermined movement of a single degree of freedom at a time. Furthermore, only one researcher group has validated their system in real-time conditions with stroke patients (Guo et al., 2022). In this study we assess the feasibility, usability and functionality of an hBMI system and training protocol for stroke rehabilitation. The system allows the continuous control of a 7-DoF upper limb exoskeleton. This is achieved by combining the continuous output of a binary electroencephalography (EEG)-decoder and a continuous EMG decoder, with a gating control flow that ensures the functional activation of both central and peripheral structures for the movement execution. Six chronic and severely impaired stroke patients without active finger extension trained functional tasks such as grasping, pointing and wrist rotations in combination with reaching movements with the proposed rehabilitation system for 10 days. The usability and functionality of the system and the training protocol were evaluated by means of feedback questionnaires. Furthermore, the outcome of this rehabilitation pilot intervention was assessed with behavioral and electrophysiological signal measurements.

## 2 Materials and methods

### 2.1 Patients

Six patients were recruited via advertisements at the University Hospital of Tübingen and physiotherapy clinics in Tübingen, Germany and via public information with stroke associations. All of them gave written consent to the procedures as approved by the ethics committee of the Faculty of Medicine of the University of Tübingen, Germany.

All participants fulfilled the following criteria: 1) paralysis of one hand with no active finger extension; 2) time since stroke of at least 9 months; 3) age between 18 and 80 years. Psychiatric or neurological conditions other than stroke, cerebellar lesion or bilateral motor deficit, pregnancy and epilepsy constituted exclusion criteria. All patients were able to understand and follow instructions.

None of the patients could extend their fingers or their wrist. According to the sensibility scores of the Fugl-Meyer-Assessment (FMA) all patients felt the arm moving and half of them felt hand movements, too. A summary of the patient’s demographics and functional data are presented in Table 1.

**TABLE 1:** Summary of demographics and functional data (*combined hand and arm scores (motor part) from the modified upper limb Fugl-Meyer-Assessment (cFMA) (excluding coordination, speed and reflexes scores; Max = 54points. These values are the average of two baseline measurements that were done 2 weeks (Pre1) and right before (Pre2) the intervention) (Ramos-Murguialday et al., 2013).

Patient	Gender	Lesion side	Age	Months since stroke	Initial Fugl-Meyer Assessment (cFMA) score*
1	M	R	65	84	9.0
2	F	R	63	50	9.5
3	M	R	49	92	6.0
4	F	L	40	182	14.0
5	M	L	62	9	15.5
6	M	L	54	34	10.5

### 2.2 Experimental protocol

**FIGURE 1:**
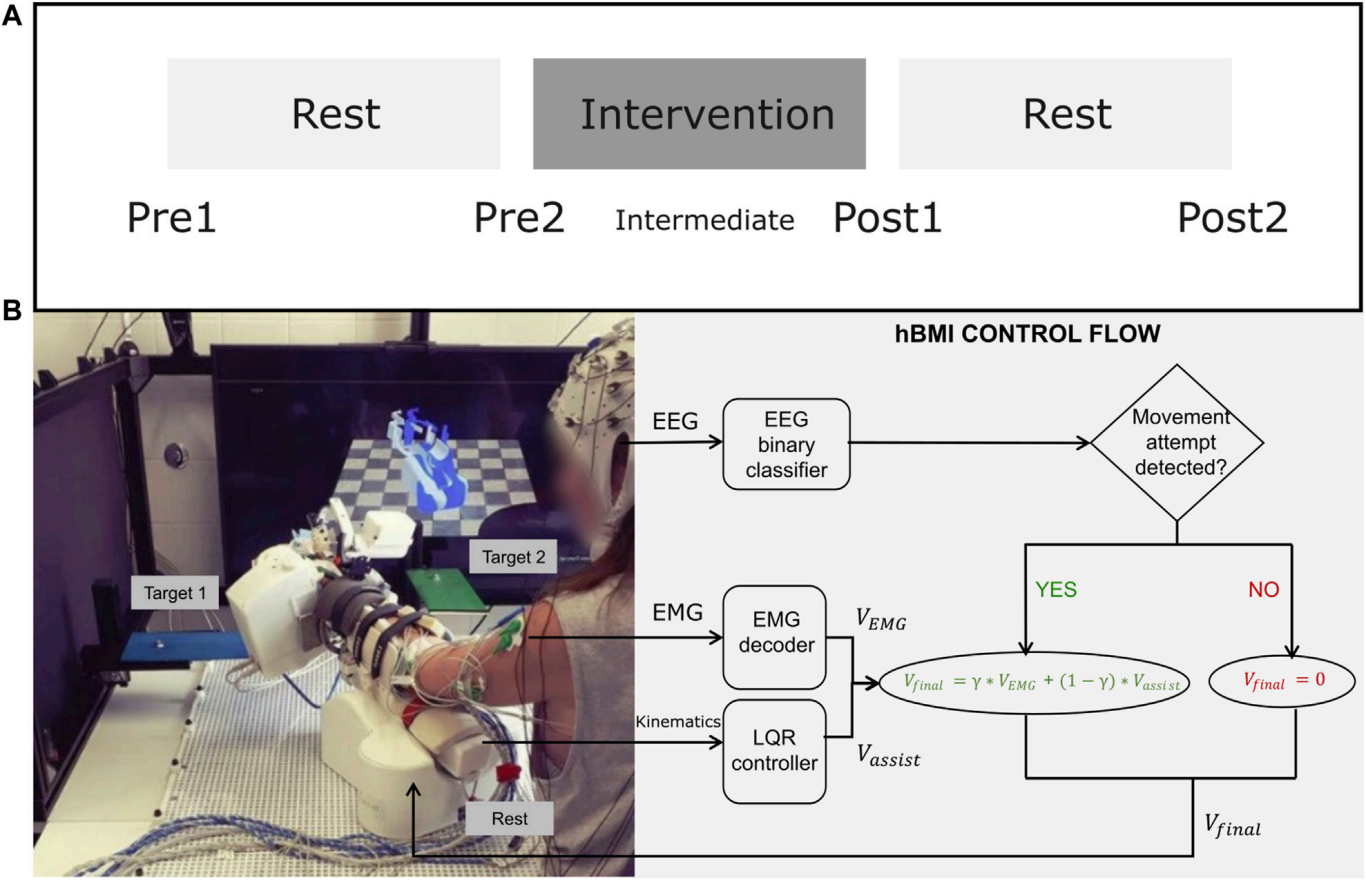
**(A)** Intervention and Assessment Schedule. **(B)**-Left: The experimental setup including EEG, EMG, the exoskeleton, the targets of two task types, and the visual feedback on the screen. An additional target was place to the right of the patient. **(B)**-Right: A diagram explaining the hierarchical control flow of the hybrid BMI.

The study consisted of 2 weeks of intervention and multiple assessment sessions (Figure 1). Patients trained daily with the rehabilitation hBMI system for 2 consecutive weeks (except Saturdays and Sundays) for 1 h, followed by an individual physiotherapy session of 30 min. Assessments included clinical scales as well as neurophysiological measurements (EMG and EEG recordings and Motor Evoked Potentials induced with Transcranial Magnetic Stimulation). These were carried out four times in total: 2 weeks before the intervention started (Pre1), once directly before (Pre2) and directly after (Post1) the intervention, and the last one 2 weeks after the end of the intervention (Post2). The two measurements taken before the intervention (Pre1 and Pre2) were averaged to establish a single baseline measurement in order to account for familiarization and test variability effects (Whitall et al., 2011). Patients rested during 2 weeks before and after the intervention. Feedback questionnaires were also administered after the first week of training (Intermediate) and right after the intervention (Post1).

Patients trained functional tasks that included coordinated reaching and hand movements towards three different target positions, which were marked with colors around the workspace. The three task types were the following:

• Forward reaching leftwards, pronation of the wrist and grasping with all fingers (red_grasp)
• Forward reaching to the front and pointing with the index finger (green_point)
• Forward reaching rightwards, supination of the wrist and opening of the fingers (blue_up)

These reaching movements always started and ended at a resting position specifically defined for each patient, in which the patient could rest comfortably.

A training session consisted of between four and six blocks. Within each block, the task types (red_grasp, green_point or blue_up) were presented in random order. Each task type or target was presented once per block. The patients had 10 s to reach each target. If the target was not reached within that time a resting break of between 3 s and 5 s followed before continuing the movement towards the target. Patients had a maximum of 4 attempts to reach the target. As soon as the target or the maximum of attempts were reached a resting break of between 3 s and 5 s followed before going back to the rest position–a movement that the patients also tried to perform actively.

The presentation of the cues indicating the task type was auditory. In addition, a screen in front of the patients showed two virtual models of the exoskeleton, a translucent one in the current position and a colored one in the target position (Figure 1).

### 2.3 Experimental setup

During the training sessions, patients sat in a chair while having their upper limb attached to a 7-DoF exoskeleton that could move and turn on a table (2D plane, DoFs 1–3), pronate or supinate the forearm (DoF 4) and extend or flex the thumb (DoF 5), the index (DoF 6) and the group of pinky, ring and middle fingers (DoF 7) (Sarasola-Sanz et al., 2015).

EEG was acquired from a 32-channel EEG cap at a sampling rate of 1000 Hz (EasyCap GmbH, BrainProducts GmbH, Germany). EMG signals were registered from surface electrodes (Myotronics-Noromed, United States) over 14 muscles of their paretic upper limb (First dorsal interosseous, Abductor pollicis longus, Extensor carpi ulnaris, Extensor carpi radialis, Extensor digitorum, Flexor digitorum superficialis, Flexor carpi radialis, Pronator teres, Biceps, Triceps, Deltoid anterior, Deltoid medialis, Teres major and Pectoralis major) at 1,000 Hz. Vertical and horizontal EOG was also recorded.

### 2.4 Hybrid brain-muscle-machine interface control

During the training with the hybrid brain-muscle-machine interface, the exoskeleton moved the patients’ upper limb based on the output of a hybrid decoder with a control flow inspired by biological principles (Figure 1) (Sarasola-Sanz et al., 2017). The EEG signal measured over the ipsilesional motor cortex was decoded and whenever the intention to move was detected (i.e., a desynchronization of the sensorimotor rhythm (SMR)), the EMG control was enabled. Then, EMG activity captured from the 14 electrodes placed on the paralyzed upper limb was continuously decoded and translated into velocity commands for each of the 7 DoFs of the exoskeleton ( $V_{EMG}$ ). To compute the final velocity sent to each of the exoskeleton DoFs ( $V_{final}$ ), the EMG velocity components were combined (Eq. 1) and weighted (γ = 0.5) with assistive velocity components ( $V_{assistive}$ ) calculated using an LQR controller, which aided the patient in reaching the target position. Hence, patients received visual and proprioceptive feedback from the movement of the exoskeleton in real-time, establishing a hybrid closed-loop control.

$$V_{final}=\gamma *V_{EMG}+\left(1-\;\gamma \right)*V_{assistive}$$ (1)

The assistive component was introduced to compensate for possible occasional mechanical issues in the exoskeleton (e.g., wheels sliding) and for the complexity that controlling a mirror myoelectric decoder might pose for patients. Our mirror decoder does not aim to achieve the highest possible performance but instead, it provides patients with feedback about their incorrect activation patterns (Sarasola-Sanz et al., 2018). Hence, a certain level of assistance was introduced to avoid frustration, especially at the beginning of therapy when users are not yet accustomed to the system and have not learned the myoelectric mirror map. The assistance level (γ = 0.5) was chosen based on previous experiments (Sarasola-Sanz et al., 2022) to ensure patients would achieve successful control of the multi-DoF interface while allowing them to learn and adapt to the myoelectric mapping over time.

To achieve a smoother and more stable control experience, the movement of the exoskeleton was not triggered (or stopped) until the EEG-decoder output was classified five consecutive times as “Movement” (or “Rest”) (Ramos-Murguialday et al., 2013). Additionally, the exoskeleton remained blocked during the rest and preparation periods, ignoring the outputs of the decoders.

### 2.5 EEG calibration and decoding methods

To choose the electrodes and frequency bands that could best control the interface, an EEG screening was introduced before the beginning of the hBMI training. During the screening patients were asked to either relax or try to open and close their paretic hand, guided by visual and auditory cues.

EEG signals were bandpass filtered (fourth order Butterworth filter at 5–48 Hz), down-sampled to 100 Hz and spatially filtered with a Surface Laplacian transform. An automated EOG artifact rejection method was applied following the method described by (Schlögl et al., 2007). Spectral estimation was performed by modelling the resulting signals as an autoregressive process of order 20 using 0.5 s-long windows and a step size of 50 ms. To identify the EEG channel and frequency bands showing the largest power difference between rest and movement, the mean power spectral density was computed and a visualization of r-squared values of these power values was created. Two raters visually selected the two most discriminative perilesional electrodes and frequency bands that would be used as input features for the decoder during the real-time hybrid control.

During real-time hybrid control, the EEG decoder was retrained at the end of each trial based upon the last 2 minutes of data collected from each condition (“Rest” and “Movement”). This adaptive strategy helped to mitigate potential effects of impedance changes or other intra-session variabilities.

### 2.6 EMG calibration and decoding methods

EMG data was filtered (10–500 Hz and 50 Hz comb filter). Five time-domain features (Mean of absolute value, Variance, Waveform Length, Root-mean-square error, and the Logarithm of the Variance) were extracted from the 14 EMG channels and normalized to zero mean and unit variance using the mean and standard deviation computed on the last minute of EMG data during the real-time hBMI operation.

The EMG activity of the patients was decoded using a mirror decoder approach (Sarasola-Sanz et al., 2018). The EMG of the same 14 muscles on the patient´s healthy arm was recorded during an initial calibration session while performing the same type of movements with the exoskeleton. This data was mirrored and processed before training the patient-specific mirror decoder. A ridge regression algorithm with a regularization parameter λ = $10^{4}$ was utilized to build the mirror decoder, which interpreted the EMG activity from the paretic arm during the real-time hBMI control. Hence, the mirror decoder enabled patients to relearn non-compensatory muscle activation patterns from their healthy arm.

### 2.7 Physiotherapy

Immediately following the hBMI training session, patients participated in a 30-min behavioral physiotherapy session tailored to their individual abilities and needs. The primary objective of these sessions was training arm and hand movements to facilitate the translation of possible gains from the hBMI training into functional motor benefits. This involved a diverse range of exercises, such as forward reaching to enhance shoulder flexion and extension, forearm supination and pronation, and elbow and wrist flexion and extension. These exercises were integrated into meaningful everyday tasks specific to each patient, such as grasping objects and bringing them closer to the body, practicing hand opening and closing to improve object manipulation skills, holding and releasing different shaped and sized objects and bringing the hand to the mouth for enhanced eating abilities.

### 2.8 Assessment

#### 2.8.1 Usability and functionality

Three questionnaires (see templates in Supplementary Material) comprising a total of 52 statements were presented to the participants to gather their feedback about the usability and functionality of the system and their expectations of the intervention. Two of them were administered in the middle (Intermediate) and right at the end of the trial (Post1), and the third one was only given right at the end (Post1). Patients were asked to indicate their level of agreement to each statement on a range from 0 (strongly disagree) to 7 (strongly agree). The statements were grouped into the following categories to simplify the analysis:

• *Exoskeleton functioning*: evaluated whether the exoskeleton moved smoothly and at a comfortable speed.
• *Exoskeleton ergonomy*: comprised statements about how comfortable it was to wear the exoskeleton.
• *Exoskeleton control*: assessed how difficult it was for participants to control the movement of the exoskeleton with their EMG and EEG activity.
• *Feedback accuracy*: evaluated the perception of the participants about the feedback provided (i.e., whether they felt that the exoskeleton moved according to or against their will).
• *Protocol design, tasks and instructions*: looked for the opinion of the participants regarding how tired they were after the training, whether the pauses were long enough, whether the tasks trained were adequate and the instructions were clear.
• *Experimenter*: analyzed participants’ opinion about the people running the experiment, whether they were experienced, and the treatment received was adequate.
• *Performance perception and expectations of improvement*: evaluated participants’ perception of how well they were performing and their expectations for the outcomes they would achieve from the intervention.

First, the answers of the two questionnaires administered in the Intermediate and Post1 measurements were compared to see if the feedback varied along the trial. Additionally, the answers to the three questionnaires at Post1 were studied to evaluate the general perception of the participants about the intervention in the aforementioned categories.

#### 2.8.2 Primary behavioral outcome measure: Fugl-Meyer assessment (cFMA)

The combined hand and arm scores (motor part) from the modified upper limb cFMA (Ramos-Murguialday et al., 2013) were used to measure the behavioral outcome (maximal score = 54 points). Patients could not perform the movements required for the scores related to coordination and speed. The scores related to reflexes add uncertainty to the measurement (Crow and Harmeling-Van Der Wel, 2008). Both types of scores were thus excluded.

#### 2.8.3 Secondary behavioral outcome measures: Broetz scale

The Broetz scale is a validated instrument specifically designed for reliable assessment of hand function in severely paralyzed stroke patients (Broetz et al., 2014). It allows to assess small variations in hand function in this group of patients using seven tasks of daily life with the paralyzed hand. It was applied right after the cFMA score in each assessment session.

#### 2.8.4 Further outcome measures based on neurophysiological data

##### 2.8.4.1 Electromyography during compliant movements

During the assessment sessions, compliant movements were recorded with the exoskeleton on the healthy (2 sessions) and paretic (3 sessions at Pre, Post1 and Post2) limbs, separately, in patients 2–6. The exoskeleton moved the upper limb of the patient towards the three predefined target positions fully automatically. The patients were asked to follow the movement of the robot without applying any counteracting force. Each session comprised five blocks, with approximately 12 trials per block (3 trials per task type). EMG activity from the 14 muscles described in Section 2.3 was acquired. In this manner, we anticipated acquiring consistent EMG recordings during assisted movements on the patients’ limbs across all assessments, ensuring comparability.

EMG data was acquired at 1 kHz. Raw data was band-pass filtered between 5and 300 Hz using a zero-phase FIR filter, full wave rectified, and low-pass filtered at 0.3 Hz (second order, Butterworth) to extract a smoothed signal envelope. After visually inspecting the EMG data, the data from the first dorsal interosseous muscle was excluded from this analysis due to high levels of noise observed in the acquired signals from this superficial muscle in most of the sessions and in most patients. The EMG data was segmented into trials from the time of the auditory signal that announced the start of the movement (‘go’ instruction) to the end of the movement (encompassing both the forward and return to the rest position phases) and aggregated into task-specific datasets. S1 Patient 1 (P1) was not included in this analysis because the motor tasks recorded in the assessment sessions were different, and therefore not comparable between them. Every EMG trial was time-normalized to 1,000 and Z-score-normalized in amplitude. For each patient, session and task, trials displaying EMG activity levels outside the range of the averaged task-specific pattern of activity ±3 units at any timepoint during the trial were excluded. Muscles with fewer than 5 remaining trials after trial rejection were not considered to exhibit a representative and stable EMG pattern associated with the assisted robotic movement. Therefore, they were not evaluated for that specific task, session, and patient. With the remaining trials, the average EMG pattern of each muscle was calculated for every patient, assessment session and task.

The Pearson correlation coefficient (CC) and the variance accounted for (VAF) metrics were utilized to evaluate the degree of similarity between two EMG profiles (Irastorza-Landa et al., 2021). Values close to 1 indicate perfect temporal coactivation for CC and a perfect match in normalized EMG pattern amplitudes for VAF. These metrics were first computed by comparing homologous EMG profiles from the two recording sessions on the healthy limb for each patient, task and muscle. This provided reference mean and standard deviation values of these metrics. Values below 1 might be attributed to inter-session variability, even in healthy datasets.

CC and VAF were subsequently computed between the averaged healthy reference EMG patterns (data from both healthy limb sessions pooled together) and the corresponding measures in the paretic limb for each patient, assessment session, task and muscle. Finally, the mean and standard deviation of CC and VAF were computed across muscles and tasks for each patient and session.

##### 2.8.4.2 Motor evoked potentials

The efficacy of cortico-muscular connections was evaluated through motor evoked potentials (MEPs) using transcranial magnetic stimulation (TMS). Having a metallic implant was an exclusion criterion that resulted in two patients (patients 2 and 3) not being eligible for this measure.

We used a magnetic stimulator (Magstim Rapid2, Magstim Ltd., United Kingdom) equipped with a figure-of-eight coil (D70 Air Film Coil, Magstim Ltd., United Kingdom) to provide single pulse stimuli over the ipsilesional hand-motor cortex of the participants. The scalp of each participant was registered with a neuronavigation system (Localite GmbH, Germany) that allowed accurate tracking of the stimulation coil relative to the hand-motor cortex. Two distal muscles of the forearm, the first dorsal interosseous (FDI) and the abductor pollicis longus (APL)) were defined as target muscles for measuring MEPs, as they allow for the evaluation of the effectiveness of the entire upper limb, from the brain to the muscles that control hand functionality. We adapted the amplitude criteria typically used to define the resting motor threshold (RMT) to prevent potential discomfort or headache (Groppa et al., 2012). The resting RMT was thus defined as the minimum stimulation intensity required to elicit at least 5 evoked potentials in the target muscles larger than 20 μV out of 10 consecutive stimulation pulses.

For the evaluation of the MEPs, we initially identified the spot that produced the largest MEPs in the target muscle (referred to as the “hot spot”) and marked this location in the neuronavigation system. Then, in a clockwise direction, we recorded a spiral mapping of the surrounding area, which was divided into a grid with a mesh-density of 1 cm and a size of 10 × 10 cm in the 3D head model. In each assessment session, 10 stimulations were delivered on each point of the grid at 120% of the RMT every 5–5.5 s. The spiral mapping concluded when less than 5 MEP responses out of 10 consecutive stimulations were obtained.

The raw EMG activity of the muscles was initially processed by removing the signal within 10 ms before and after the stimulation artifacts and correcting for any voltage differences at the edges of the merged traces. The resulting signal was band-pass filtered between 10 and 2000 Hz and notch filtered using a second order Butterworth filter. Then, the signal was trimmed down to 200 ms trials from −100 to 100 ms with respect to the stimulation trigger. To avoid a facilitation effect of EMG contraction before the stimulation trigger on the MEPs, we implemented an automatic method for muscular activity detection. The envelopes of the EMG signal of 10 ms sliding windows with 1 sample step were calculated in the interval prior to the stimulation artifact [-100 0]ms. The envelope values of each sliding window were averaged, and the mean and standard deviation between trials recorded in the same stimulation spot and channel were used to calculate the rejection threshold. Those trials containing at least one sliding window value that exceeded 4 standard deviations above the mean were discarded. Clean trials were pooled together and averaged for each patient, channel, and spot in the spiral mapping of the motor cortex. The peak-to-peak amplitude and latency of the resulting potential were computed.

#### 2.8.5 hBMI control performance

Performance of patients controlling the hBMI system were evaluated using the EEG decoder outputs. The EEG decoder computed an output every 25 ms and it was positive if the brain state was classified as movement intention. The EEG performance measure is represented by the percentage of positive outputs out of all decoder outputs. Only the first try to reach each target was considered to evaluate values comparable between patients. This measure serves as an estimation of the ability of the patients to operate the system.

## 3 Results

### 3.1 Usability and functionality

#### 3.1.1 Intermediate-Post1 feedback comparison

**FIGURE 2:**
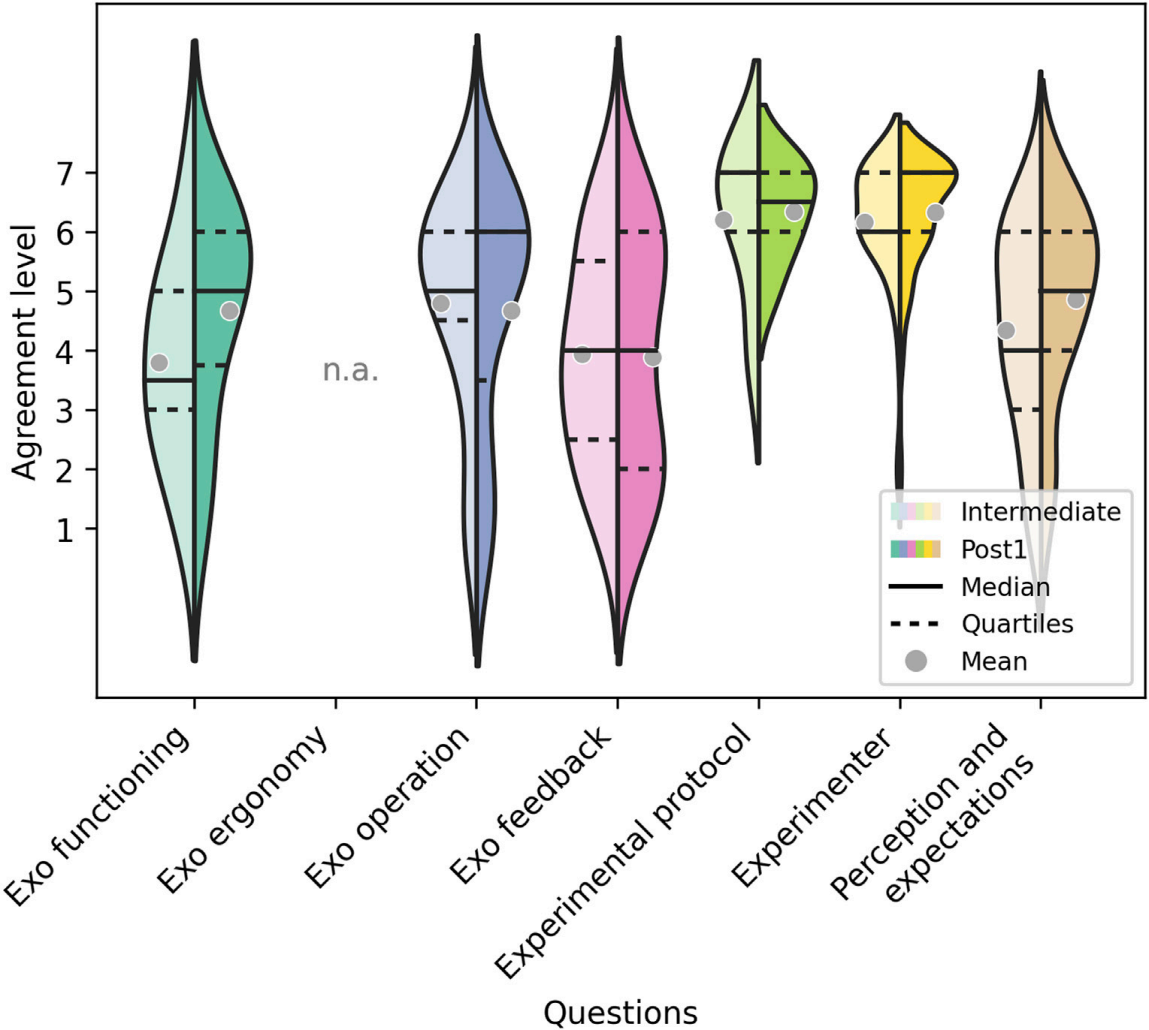
Responses of the participants to the questionnaires filled out in the middle (Intermediate) (Left half) and at the end of the intervention (Post1) (Right half) for each of the 7 categories. The horizontal lines define the median values and the gray dots the mean values.

Figure 2 represents the mean, median and the distribution of the responses of the participants to the questionnaires filled out in the middle (Intermediate) and at the end of the intervention (Post1). It shows satisfactory mean response values (in the range [3.8, 6.2]) in the middle of the intervention, which became even better in most of the categories after the intervention (mean values in the range [3.9, 6.3]). Median values either remained stable or improved in the Post1 measurements. Furthermore, the median values at Post1 fell within the range of [5,7] in all the categories but the “Exo feedback”, where the median was 4.

#### 3.1.2 Post1 feedback evaluation

**FIGURE 3:**
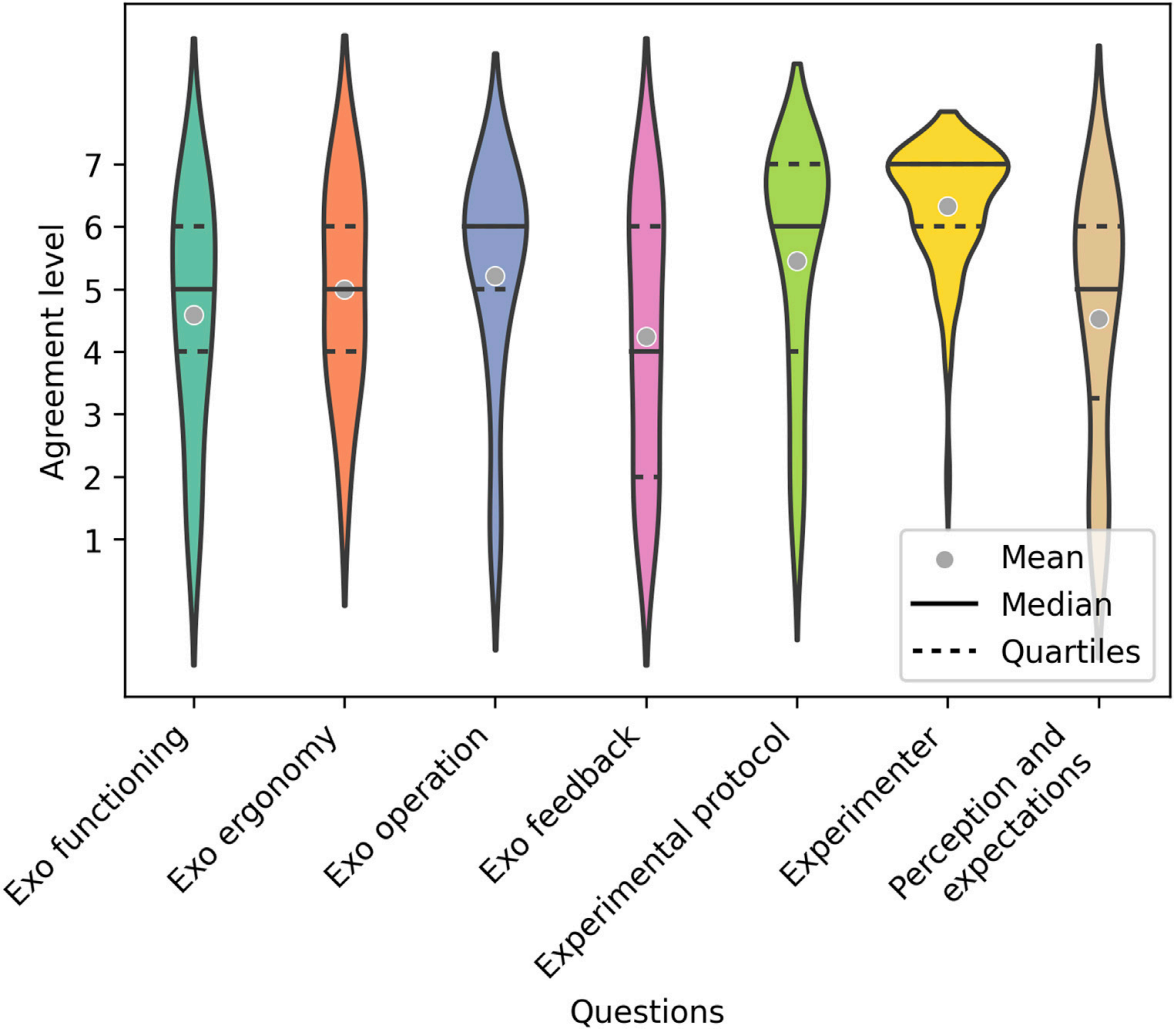
Responses of the participants to the questionnaires filled out and at the end of the intervention (Post1) for each of the 7 categories. The horizontal lines define the median values and the gray dots the mean values.

The answers to the three questionnaires at the end of the trial are illustrated in Figure 3, which show positive feedback of the system’s usability and functionality as well as of the users’ expectations. Mean values lie within [4.24, 6.32], and the distribution of the responses was skewed towards higher values, as reflected by the median values of 5 or higher for all categories but the " Exo feedback".

### 3.2 Primary behavioral outcome measure: Fugl-Meyer assessment (cFMA)

The changes of the Fugl-Meyer scores of arm and hand combined for the intervention ranged from −0.5 to +3 points with mean and standard deviation of 0.83 and 1.3 (Table 2). There was no statistical difference of the scores to 0 (t-test). Remarkably, patient 5 showed a three-point improvement on the scale after only 10 days of training.

**TABLE 2:** Changes of the Fugl-Meyer scores (arm and hand combined) and the Broetz scores during the intervention.

Patient	cFMA	Broetz scale
1	0.0	2.5
2	1.0	1
3	−0.5	3
4	0.0	4
5	3.0	12
6	1.5	−2

### 3.3 Secondary behavioral outcome measures: Broetz scale

The changes of the Broetz scores ranged from −2 to +12 points with mean and standard deviation of 3.4 and 4.7 (Table 2). There was no statistical difference of the scores to 0. The notable improvement of Patient 5 is also reflected on the Broetz scale, representing an advancement of multiple steps of complexity in the tasks performed during the test.

### 3.4 Further outcome measures based on neurophysiological data

#### 3.4.1 EMG during compliant movements

**FIGURE 4:**
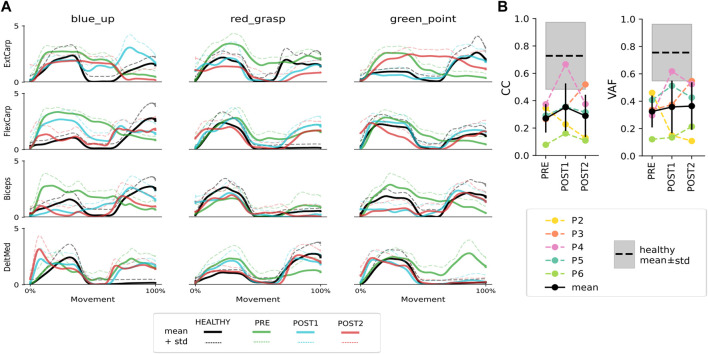
**(A)** Average EMG activations (solid lines) + 1 standard deviation (dashed lines) of a subset of four muscles in the combined healthy reference datasets (black) and in the paretic limb in Pre (green), Post1 (blue) and Post2 (red) assessment measurements in a representative participant. **(B)** Colored lines represent the CC and VAF values averaged among muscles and tasks for each patient in different assessment sessions. The mean and standard deviation values among all patients (P2-P6) are indicated by a black errorbar. The mean and standard deviation of the healthy reference are represented by a dashed black line and a gray shaded area, respectively.

The task-specific normalized and averaged EMG profiles of four muscles in Patient 4 are depicted for both the healthy and the paretic limbs in various assessment sessions in Figure 4A (See Supplementary Material for the rest of the patients). In the case of this patient, it can be observed that the task-specific EMG profiles after the intervention (Post1, blue) became more similar to those recorded in the healthy limb (Healthy, black), particularly for certain muscles (e.g., Extensor carpi ulnaris, Flexor carpi radialis, Biceps, and Deltoid Medialis during the green_point task). In some cases, no significant changes in muscle modulation were observed across different time points (e.g., Biceps or Flexor carpi radialis during the red_grasp task).

In general, most patients exhibited CC and VAF values below the average healthy reference for all or some of the tasks, indicating at least a partial mismatch in temporal coactivation and EMG patterns between limbs (see Figure 4B). However, the mean VAF value demonstrates an upward trend from Pre to Post2, and the CC values show an increase in Post1. When examining the results for each patient individually, it can be observed that Patient 2 showed the best CC and VAF values at the baseline (Pre), with a significant decrease at both timepoints after the intervention (Post1 and Post2). However, the remaining four patients showed an increase or similar CC and VAF values after the intervention (Post1) compared to the baseline status (Pre).

#### 3.4.2 Motor evoked potentials

**FIGURE 5:**
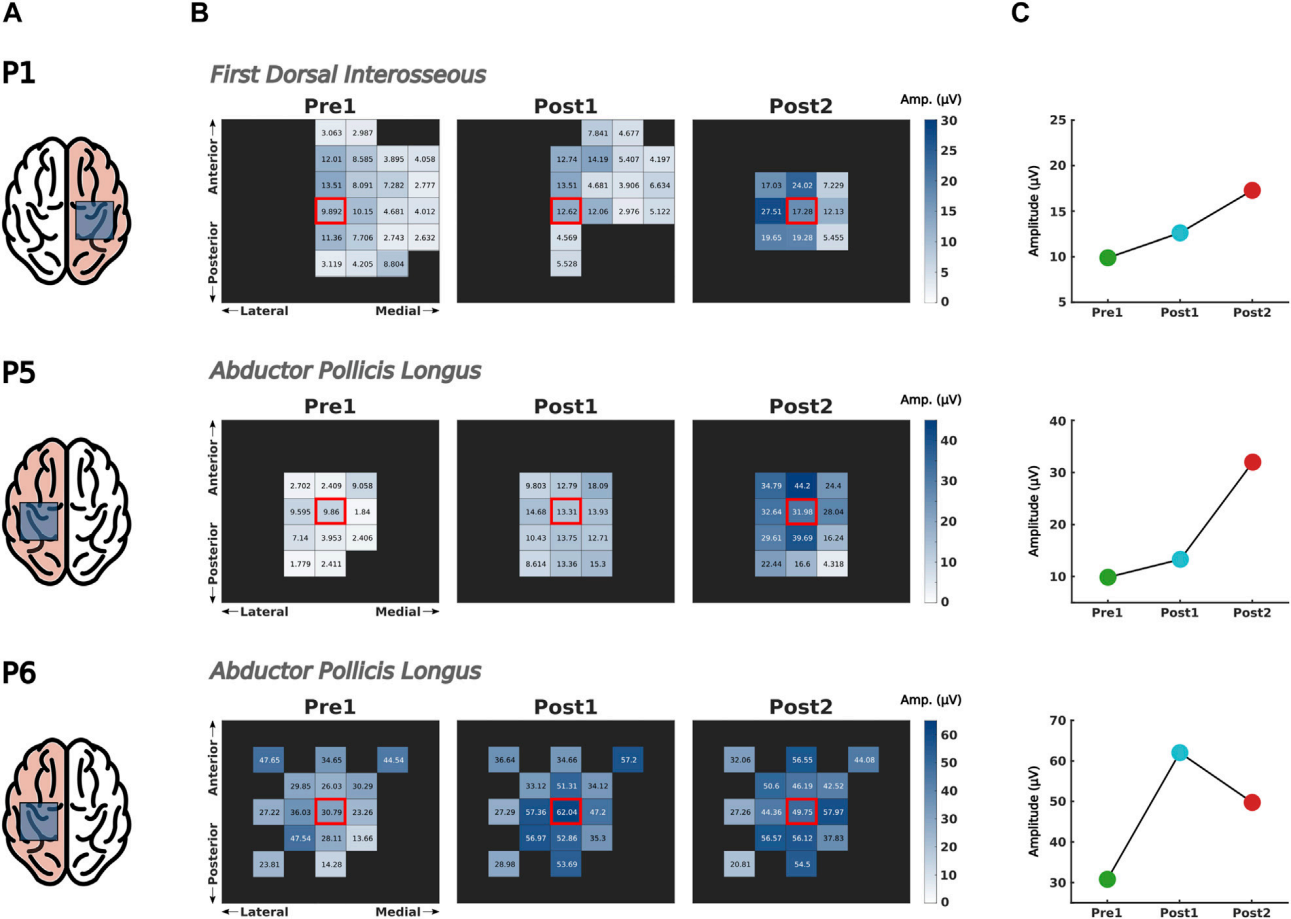
Comparison of corticospinal excitability over time. **(A)** Corticospinal excitability was assessed by means of motor evoked potentials (MEPs) over the hand motor cortex of the ipsilesional hemisphere, represented inside the shaded square in the highlighted brain laterality. **(B)** The peak-to-peak amplitude of 10 MEPs elicited in the target muscle were measured in the spot presenting larger muscular response (i.e., hot spot, indicated with a red square) and in the surrounding cortical region. **(C)** Changes in MEP size of the hot spot before and after intervention.

Figure 5 shows the results of the hand-mapping around the hot spot of each participant along the different assessment sessions (see Supplementary Material for the stimulation intensity utilized for each participant). No motor evoked potentials were obtained in Patient 4 in any of the measurements.

Patient 1 exhibited a significant increase of MEP amplitude in the FDI muscle right after finishing the intervention (Post1) and continued increasing even 2 weeks after (Post2). Furthermore, the region adjacent to the hot spot presented larger MEP responses, suggesting an improvement of synaptic efficacy of corticospinal tracts (Figure 5, upper row).

For Patient 5, the corticospinal efficacy of the APL increased immediately after completing the intervention (Post1) and continued to rise during the 2 weeks following this phase (Post2) (Figure 5, middle row). The excitatory effect of the intervention was observable not only at the hot spot but also in the surrounding area, as depicted in the heatmaps.

As shown in the heatmaps of Patient 6 (Figure 5, bottom row), the corticospinal tracts projecting to APL from neighboring cortical areas exhibited stronger responses following the intervention (Post1) and this effect persisted for up to 2 weeks (Post2). When focusing on the hot spot, an enhancement of synaptic efficacy was also observed over time.

### 3.5 hBMI control performance

**FIGURE 6:**
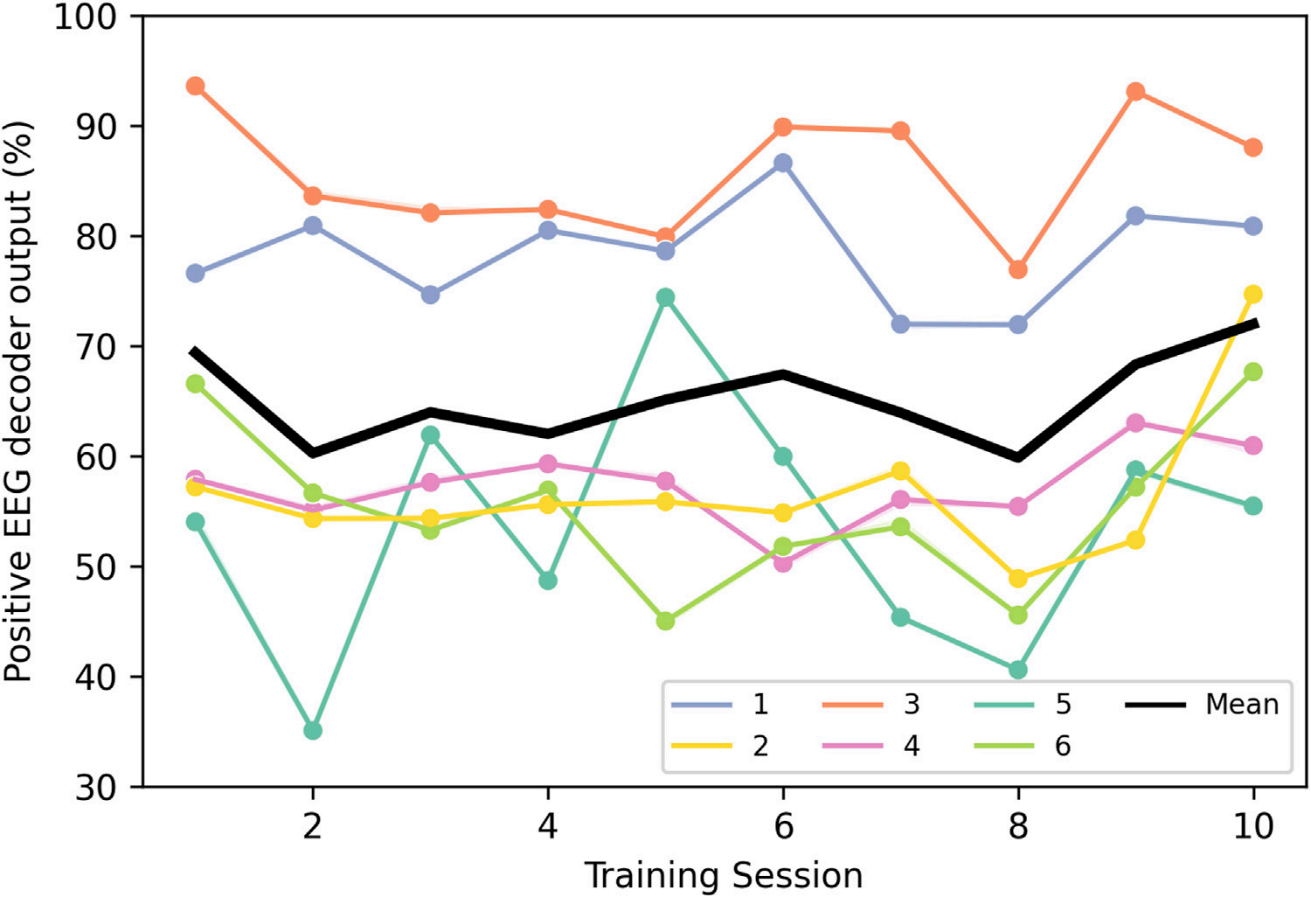
Percentage of EEG decoder outputs that were decoded as “Movement” during the trial time for each patient (1–6) and training session, as well as the mean of all of them.

All the patients, despite being severely impaired could control the hBMI successfully to perform coordinated functional arm and hand tasks. The percentage of positive outputs of the EEG decoder is shown in Figure 6. For most patients, the performance stayed on a similar level throughout the intervention (*p*-values >0.05 for all linear regressions). Only Patient 5 showed larger variability than the other patients. For a major part of the intervention the performance was in a range of 60%–70% on average. There are only very few sessions for Patients 2, 5 and 6 during which performance dropped below 50%. Patients 1 and 3 achieved the highest performance values with average values between 75% and 95%.

## 4 Discussion

In this pilot trial we demonstrated the feasibility of an intensive rehabilitation training with a hybrid interface that integrates muscle and brain activity to control an exoskeleton with 7 DoFs. The feedback received from the 6 patients demonstrates the usability and functionality of the system, as well as the confidence of the participants about its operation and its potential for rehabilitation. Furthermore, we showed the therapeutic potential of the system in severely paralyzed and chronic patients, who are not eligible for most of the existing and clinically available treatments.

The patients’ responses to the questionnaires show that they highly value the system, the protocol and the experimenters. The responses at the Intermediate measurement indicate that even severely affected and chronic patients, after just 5 days of use, can adapt to the hybrid interface and are satisfied with its operation. This is further demonstrated by the brain activity decoding performance values, which range from 60% to 70% on average, showing that from the beginning, patients were able to adapt to the EEG decoder to successfully control the interface, even though it was retrained after each trial. Moreover, the performance remained consistently stable throughout the intervention for all patients except Patient 5, who exhibited a greater degree of variability. However, it is important to note that Patient 5 also demonstrated the most significant improvement on the Fugl-Meyer scale. This suggests that higher variability or a lower percentage of positive outputs from the EEG decoder did not hinder the patient’s recovery process, as other variables, such as the myoelectric control of the interface, also influence the rehabilitation outcome. The EMG decoding performance was not included in these analyses as the goal of the mirror myoelectric decoder in our system is not to achieve the highest performance possible, independently of whether patients produce correct or pathological activations. Instead, its purpose is to offer a reference map of healthy EMG activity that could help patients correct their abnormal patterns as a means for rehabilitation. Nevertheless, performance values of the mirror myoelectric decoder method in various configurations can be found in (Sarasola-Sanz et al., 2018).

Comparing the ratings between the Intermediate and Post stages reveals an increase: patients improve their perception of the “Exo functioning” and “Exo operation”, as well as their “Performance perception and their expectations of improvement”. This implies that patients became more comfortable operating the interface and felt that they were controlling it better over time. Additionally, they gained more confidence in the system’s potential to assist in their recovery.

In the responses to the final questionnaires, patients gave a positive evaluation of the ergonomics of the exoskeleton. Extrapolating from these results on a prototypical research tool, we expect this evaluation to be even more positive once the current system is industrialized for use in routine clinical settings.

The values for “Exo feedback” are the lowest among all the question groups. However, they have an average value above 4, which is very positive considering the complexity of the hybrid feedback with the mirror decoder. Furthermore, the system aims to provide patients with feedback about whether they are activating their muscles correctly or not, rather than simply translating muscle activations, pathological or not, into a movement towards the target. This means that although patients try to bring the exoskeleton to the target, if the brain and muscle activations were not correct, the exoskeleton would deviate from the trajectory towards the target, and therefore, patients might perceive the feedback as not going in the desired direction. However, this is the foundation on which our system is based, to promote the correction of pathological patterns and thus encourage motor learning. Additionally, it should be mentioned that half of the patients had compromised proprioceptive sensation in the hand, and two of them also in the arm. These patients might not have been able to distinguish the direction in which their limb was being moved without relying on visual feedback. This could have also affected their perception and evaluation of the exoskeleton’s movements.

Half of the patients show improvements of arm function after a 2-week training, according to the Fugl-Meyer scale. Even a small enhancement on the Fugl-Meyer scale could lead to significant changes in behavior, particularly for patients who are severely and chronically paralyzed and are not expected to experience any spontaneous improvements in their behavior. Patients who did not exhibit improvement could potentially be classified as hBMI non-responders, a phenomenon documented in previous research (Vidaurre and Blankertz, 2010). Another possibility is that the intervention might have been too short to induce changes capturable by this clinical scale. Nevertheless, it is worth noting that the sensitivity of the scores in this severely paralyzed patient group might be insufficient. The Broetz scale was included to capture finer improvement in the hand joints. This scale was able to capture improvements in hand function in 5 of the 6 patients along the intervention, which were not always reflected in changes in the Fugl-Meyer scale.

Additionally, objective assessment measurements based on neurophysiological parameters were included to evaluate the rehabilitation effects. Despite being severely impaired, these patients exhibit some degree of ability to activate paretic muscles in a task-specific manner with consistent EMG activation profiles during compliant movements. Moreover, the results of the CC and VAF analyses show that the muscle activation patterns become more similar to those in the healthy arm after the intervention in 4 of the 5 patients. This reflects the positive effect of using a mirror decoder of healthy activity as a reference model, upon which the feedback (i.e., exoskeleton movement) provided to the patients is founded (Sarasola-Sanz et al., 2018). The poor results of Patient 2 might be due to the fact that this patient was constantly performing a rocking movement of the trunk during the therapy, which could have affected the consistency of the measurements. The overall CC and VAF levels of patients do not necessarily align with the FMA or Broetz values at those timepoints. However, it is important to consider that these clinical scales assess a broader repertoire of functional and analytical movements, whereas this EMG analysis focuses solely on three specific motor tasks. Lastly, CC values decrease in the follow-up measurement after 2-week of rest, indicating the influence of the training on the way patients recruit their muscles. A longer intervention would likely be necessary to sustain the improvements gained during the training over time and to proof its effect on recovery.

Previous research has demonstrated that EEG and EMG signals provide complementary information (Balasubramanian et al., 2018; López-Larraz et al., 2024). However, the relationship between brain and muscle activations remains challenging to understand and characterize. Significant advancements have been made in utilizing features that capture this relationship for the control of rehabilitation devices (Chowdhury et al., 2019; Colamarino et al., 2021; de Seta et al., 2022; Guo et al., 2022; Pichiorri et al., 2023). However, they are still limited to decoding movement attempts that trigger predefined movements and do not allow for continuous and skillful control of a multi-DoF interface. Moreover, their real-time application in stroke patients still requires a greater number of validation studies. Alternatively, we designed an hBMI with a gating strategy that follows the biologically natural motor command pathway, ensuring constant activation of both central and peripheral structures throughout the entire movement duration. Our results showed that the MEP amplitude in upper-limb muscles increased, and that the effective area of the hand-motor cortex broadened after the intervention, indicating that the hBMI training could strengthen corticospinal connections. Our hBMI is a neurorehabilitative technology that engages the entire sensorimotor network from the brain to muscles, potentially resulting in stronger muscular responses. The results we observed after the 2-week intervention lend support to the notion of enhanced corticospinal plasticity, which endures over time, due to the hybrid control of the hBMI.

Physiotherapy also influences the recovery, but in this pilot, we did not focus on disentangling physiotherapy from hBMI effects. Instead, we were testing their combined effect, following previous research (Ramos-Murguialday et al., 2013).

Altogether, the current study in severely paralyzed patients validates the feasibility, usability and functionality of this novel hBMI system, which allows the training of complex functional tasks and involves both central and peripheral structures of the nervous system establishing a biologically inspired closed-loop control. This pilot trial also indicates the potential of this hBMI system to potentiate the plasticity of the entire neural system from the brain to the muscles, which could lead to the reconnection of such structures and by extension, to motor recovery. This would have to be validated in subsequent longitudinal rehabilitation interventions with a larger number of patients, and control groups to compare the effects to those achieved with other rehabilitation methods such as physiotherapy and traditional BMIs.

## Data Availability

The raw data supporting the conclusion of this article will be made available by the authors, without undue reservation.
