# Supplementary material for: A hybrid brain-muscle-machine interface for stroke rehabilitation: Usability and functionality validation in a 2-week intensive intervention
Source: Front Bioeng Biotechnol. 2024 Apr 12;12:1330330. doi: 10.3389/fbioe.2024.1330330 (PMC11046466; doi:10.3389/fbioe.2024.1330330)
Supplement: Supplementary file 1 [file DataSheet1.docx]

Supplementary Material

# Feedback Questionnaires

## Feedback Form 1 for hBMI Training (FF1)

We would like to request your evaluation of the recent training sessions. On the following page, you will find a series of statements and questions that pertain to your mental imagery of movement, your actual physical movement, and the movements of the robot arm. With this questionnaire, we aim to assess how easy or challenging the movements were for you and how clearly you could visualize them. Additionally, we would like to know how well you believe you performed the movement and how you perceived the movements of the robot or the hand orthosis.

Please decide for each statement how much it personally applies to you. There are no right or wrong answers; please respond as spontaneously as possible. If you are unsure, please choose the answer that seems most appropriate to you.

We are solely interested in your personal impression. This survey is for research purposes only and will be analyzed anonymously.

The numbers mean the following:

① = "not at all true"

② = "not true"

③ = "not really true"

④ = "partly true"

⑤ = "rather true"

⑥ = "true"

⑦ = "completely true"

1. I understood the instructions well; there were no ambiguities.

① ② ③ ④ ⑤ ⑥ ⑦

2. I could clearly visualize the hand/arm movement.

① ② ③ ④ ⑤ ⑥ ⑦

3. I performed the movement (visualization, planning, and execution) well.

① ② ③ ④ ⑤ ⑥ ⑦

4. During the movement of the paralyzed hand, it was challenging for me not to move the unaffected hand/arm. My hand/arm muscles kept tensing up repeatedly.

① ② ③ ④ ⑤ ⑥ ⑦

5. The robot moved my arm and hand smoothly and without interruption.

① ② ③ ④ ⑤ ⑥ ⑦

6. The robot moved my arm and hand at a comfortable pace.

① ② ③ ④ ⑤ ⑥ ⑦

7. The robot consistently moved my arm and hand in the correct direction.

① ② ③ ④ ⑤ ⑥ ⑦

8. The robot executed the movements as I had imagined.

① ② ③ ④ ⑤ ⑥ ⑦

9. The robot’s movements hindered my ability to focus on the movement.

① ② ③ ④ ⑤ ⑥ ⑦

10. The robot’s movements helped me find the right strategy for the movement.

① ② ③ ④ ⑤ ⑥ ⑦

11. The robot’s movements matched the perceived performance of my movement.

① ② ③ ④ ⑤ ⑥ ⑦

12. I was able to perform the hand and arm movements effectively.

① ② ③ ④ ⑤ ⑥ ⑦

Do you have any comments or feedback?

## Feedback Form 2 for hBMI Training (FF2)

On the following page, you will find a series of statements related to the robot training. The first section focuses on assessments of the most recent training session, which is the one that occurred most recently.

The statements in the second section pertain to the week before the last training session. Please choose the answer for each statement that best applies to you. Try to respond as spontaneously and honestly as possible. There are no right or wrong answers; we are interested in your personal opinion.

Please read through all the sentences and do not skip any answers. If you are unsure, select the response that seems most appropriate to you.

For the sake of simplicity, the questionnaire uses the term “therapist” referring to the experimental leader or institute staff who assisted you during the training session. Additionally, the questionnaire uses only the male form the “therapist” instead of both forms "the therapist/the therapist (female)" or "the therapist/the therapist (male)."

This study is solely for scientific research purposes and will be conducted and evaluated anonymously.

"How much do you currently feel limited by your paralysis?

Very strongly []

strongly []

moderately []

slightly []

not at all []

The numbers mean the following:

① = "not at all true"

② = "not true"

③ = "not really true"

④ = "partly true"

⑤ = "rather true"

⑥ = "true"

⑦ = "completely true"

Section 1- In the last training session:

1. My therapist's demeanor appeared professional.

① ② ③ ④ ⑤ ⑥ ⑦

2. I noticed that my therapist possesses a high level of expertise.

① ② ③ ④ ⑤ ⑥ ⑦

3. My therapist's behavior puzzled me.

① ② ③ ④ ⑤ ⑥ ⑦

4. I felt that my therapist could understand my behavior.

① ② ③ ④ ⑤ ⑥ ⑦

5. I noticed that my therapist has extensive experience.

① ② ③ ④ ⑤ ⑥ ⑦

6. I had the impression that my therapist could empathize with my situation.

① ② ③ ④ ⑤ ⑥ ⑦

7. I felt uncomfortable.

① ② ③ ④ ⑤ ⑥ ⑦

8. I felt the need to defend myself.

① ② ③ ④ ⑤ ⑥ ⑦

9. I felt like a student.

① ② ③ ④ ⑤ ⑥ ⑦

10. I noticed that my therapist is well-educated.

① ② ③ ④ ⑤ ⑥ ⑦

11. I had the feeling that my therapist understood me.

① ② ③ ④ ⑤ ⑥ ⑦

Section 2 - In the week before the last training session:

12. During training, I saw a way to deal with my difficulties.

① ② ③ ④ ⑤ ⑥ ⑦

13. I was convinced that I would be noticeably better by the end of the training.

① ② ③ ④ ⑤ ⑥ ⑦

14. I was convinced that there would be improvements soon due to the training.

① ② ③ ④ ⑤ ⑥ ⑦

15. I had the feeling that the training would help me.

① ② ③ ④ ⑤ ⑥ ⑦

Thank you very much!

## Feedback Form 3 for hBMI Training (FF3)

We kindly request you to answer various questions in this questionnaire regarding the most recent training session. Below, you will find several statements and questions related to your experience of the movement and control of our exoskeleton. Additionally, the ergonomics of the robot are part of these questions.

Please decide, for each statement, on a scale from 1 to 7 how much it applies to you:

1: "not at all true"; 2: "not true"; 3: "not really true"; 4: "partly true"; 5: "rather true"; 6: "true"; 7: "completely true".

There are no right or wrong answers! Please respond honestly. If you are uncertain about an answer, please choose the one that best fits. We are interested in your subjective opinion.

Exoskeleton (the "robot"):

1. The exoskeleton moved without interruptions.

① ② ③ ④ ⑤ ⑥ ⑦

2. The exoskeleton moved at a comfortable speed.

① ② ③ ④ ⑤ ⑥ ⑦

3. The hand module of the exoskeleton was comfortable.

① ② ③ ④ ⑤ ⑥ ⑦

4. The arm module of the exoskeleton was comfortable.

① ② ③ ④ ⑤ ⑥ ⑦

Control difficulty:

5. The duration of one exercise ("trial") was too short.

① ② ③ ④ ⑤ ⑥ ⑦

6. Achieving the end position was too difficult and frustrating.

① ② ③ ④ ⑤ ⑥ ⑦

7. Controlling hand movement was difficult.

① ② ③ ④ ⑤ ⑥ ⑦

8. Controlling arm movement was difficult.

① ② ③ ④ ⑤ ⑥ ⑦

Tasks and Experimental Setup:

9. The tasks I performed were boring.

① ② ③ ④ ⑤ ⑥ ⑦

10. The tasks I performed were too easy.

① ② ③ ④ ⑤ ⑥ ⑦

11. The tasks I performed were helpful for practical application outside of training.

① ② ③ ④ ⑤ ⑥ ⑦

12. Reaching the target position for all movements was easy and intuitive.

① ② ③ ④ ⑤ ⑥ ⑦

13. The auditory signals were always clear both acoustically and in terms of content.

① ② ③ ④ ⑤ ⑥ ⑦

14. The tasks I performed were too difficult.

① ② ③ ④ ⑤ ⑥ ⑦

15. Some of the targets were too far away, making them unattainable.

① ② ③ ④ ⑤ ⑥ ⑦

16. Maintaining the correct posture throughout the entire experiment was tiring.

① ② ③ ④ ⑤ ⑥ ⑦

17. The training rounds (round = a set of exercises) were too long. I would have preferred more breaks.

① ② ③ ④ ⑤ ⑥ ⑦

18. I found it very difficult to concentrate on the task during the experiment due to distractions (e.g., people in the room, noises).

① ② ③ ④ ⑤ ⑥ ⑦

19. My attention waned because I did not find the tasks challenging enough.

① ② ③ ④ ⑤ ⑥ ⑦

20. The overall duration of the training session was too long, and I became fatigued at some point.

① ② ③ ④ ⑤ ⑥ ⑦

Perception of Movement Support by the Robot:

21. I had the impression that the exoskeleton supported the movements I performed (or wanted to perform) well.

① ② ③ ④ ⑤ ⑥ ⑦

22. I had the impression that the exoskeleton countered the movements I performed.

① ② ③ ④ ⑤ ⑥ ⑦

23. The exoskeleton always moved in the correct direction.

① ② ③ ④ ⑤ ⑥ ⑦

24. The exoskeleton executed the movements as I had imagined.

① ② ③ ④ ⑤ ⑥ ⑦

General Explanations:

25. I fully understood the explanations provided by the experiment leaders. There was no point at which I was uncertain.

① ② ③ ④ ⑤ ⑥ ⑦

Room for Comments:

# Supplementary Figures and Tables

## Supplementary Figures

**P2**

**P3**

**P4**

**P5**

**P6**


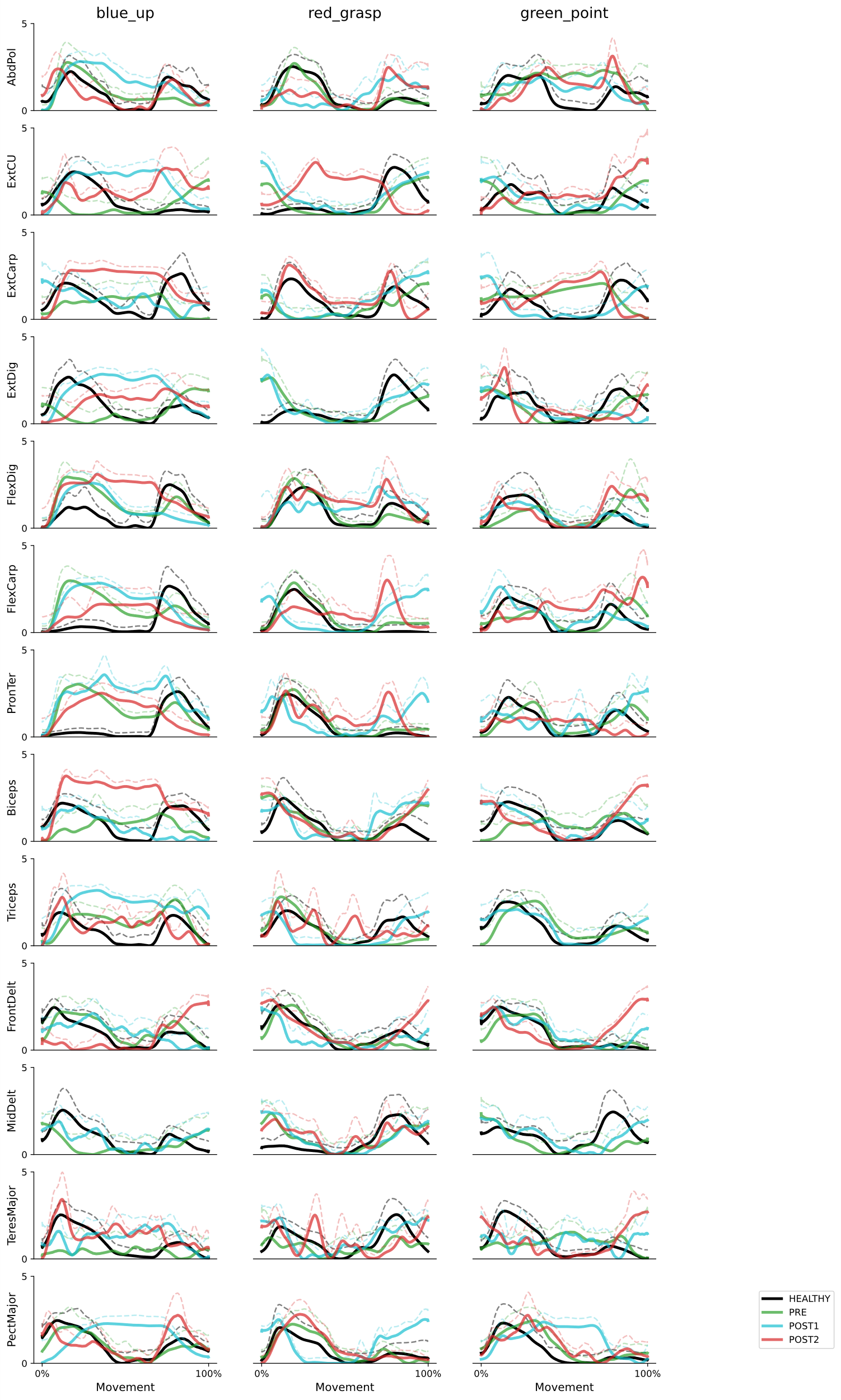

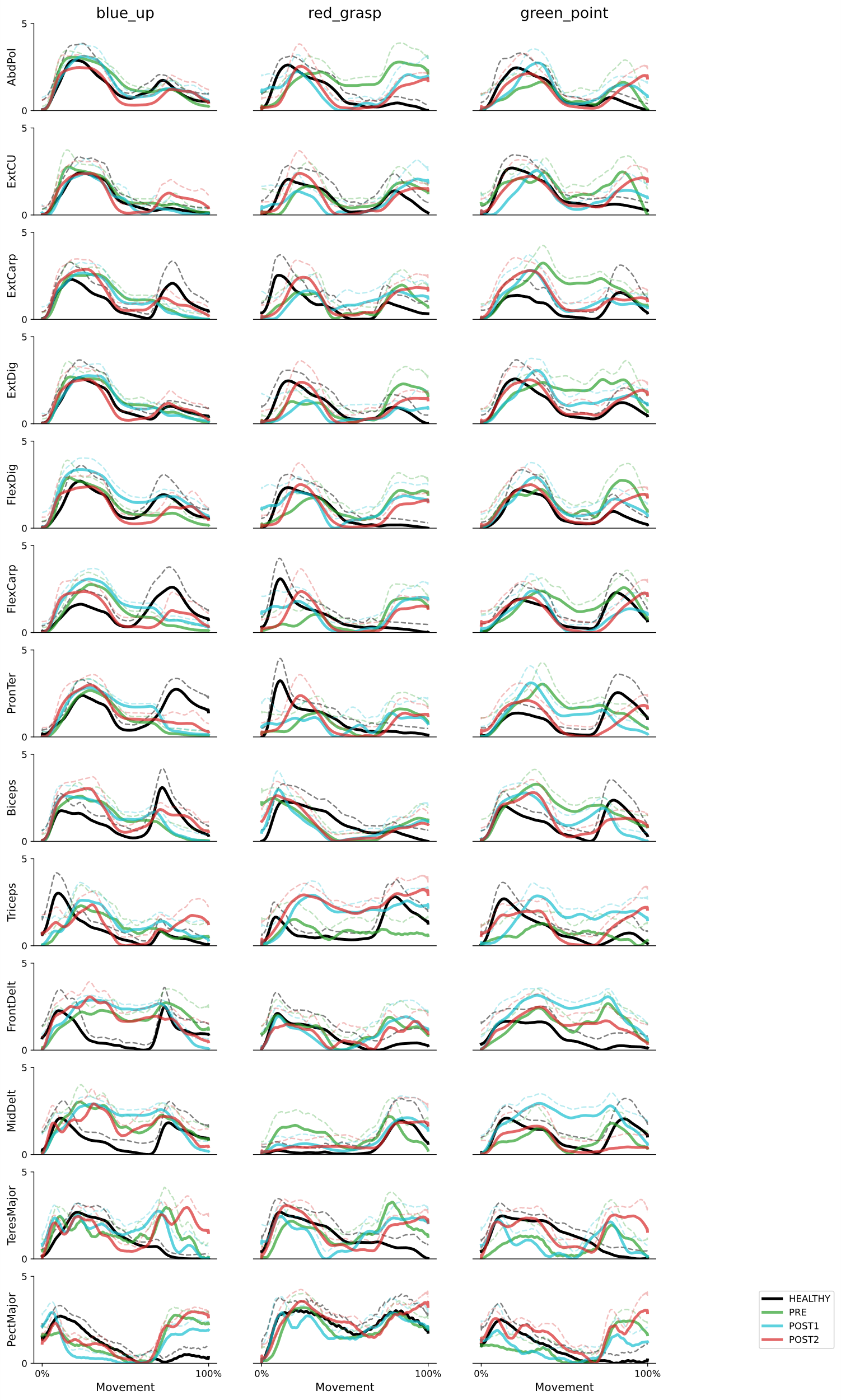

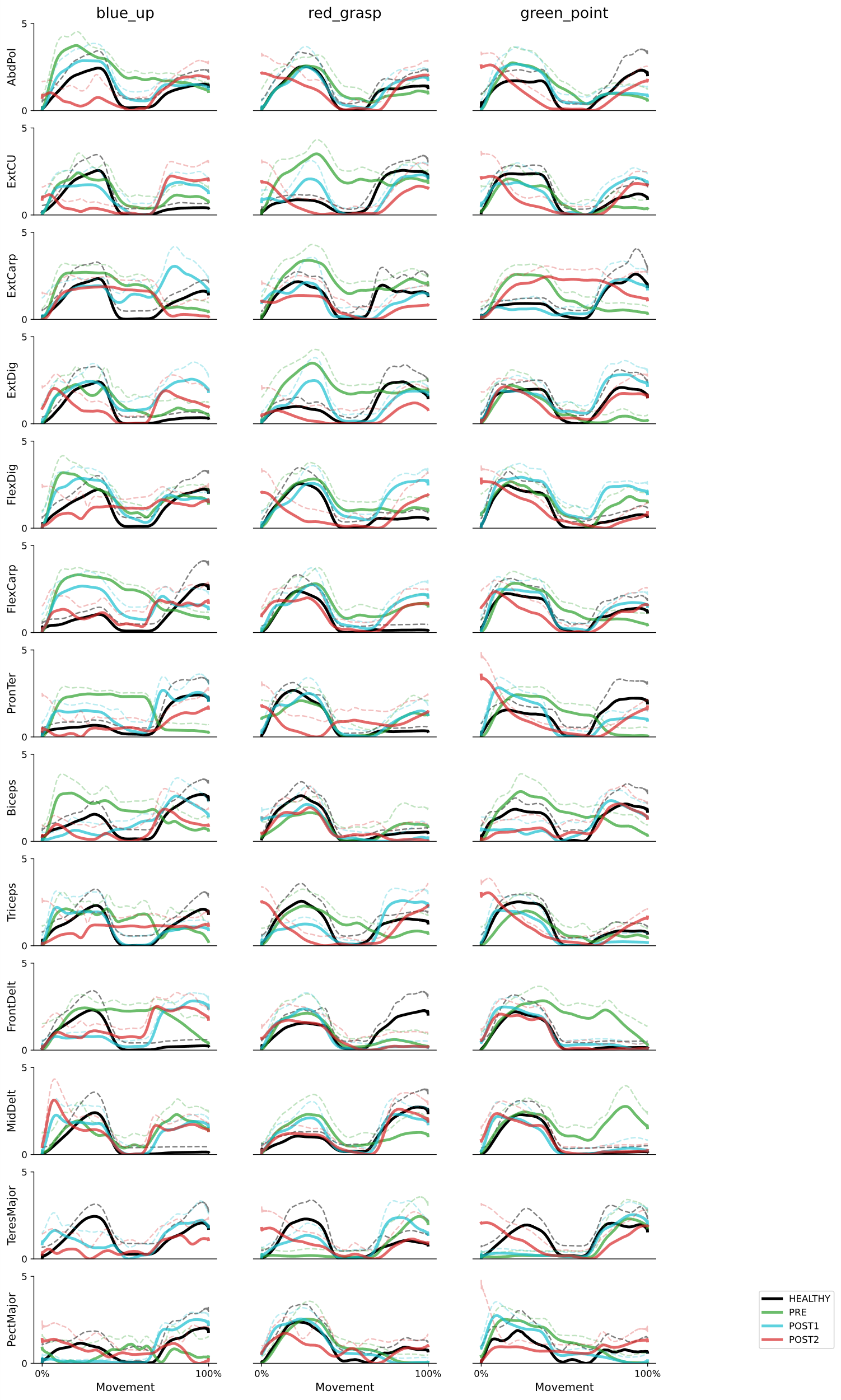

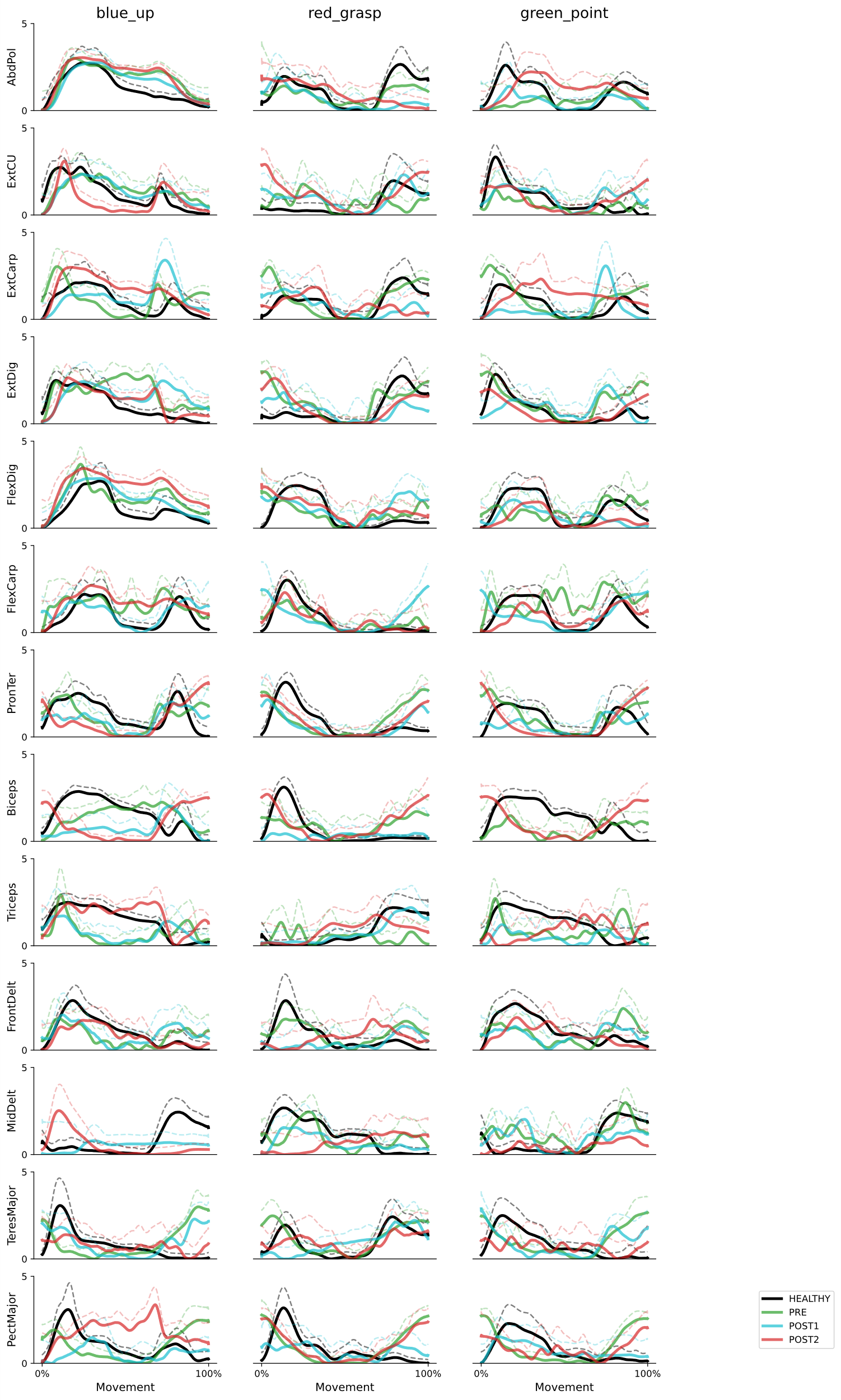

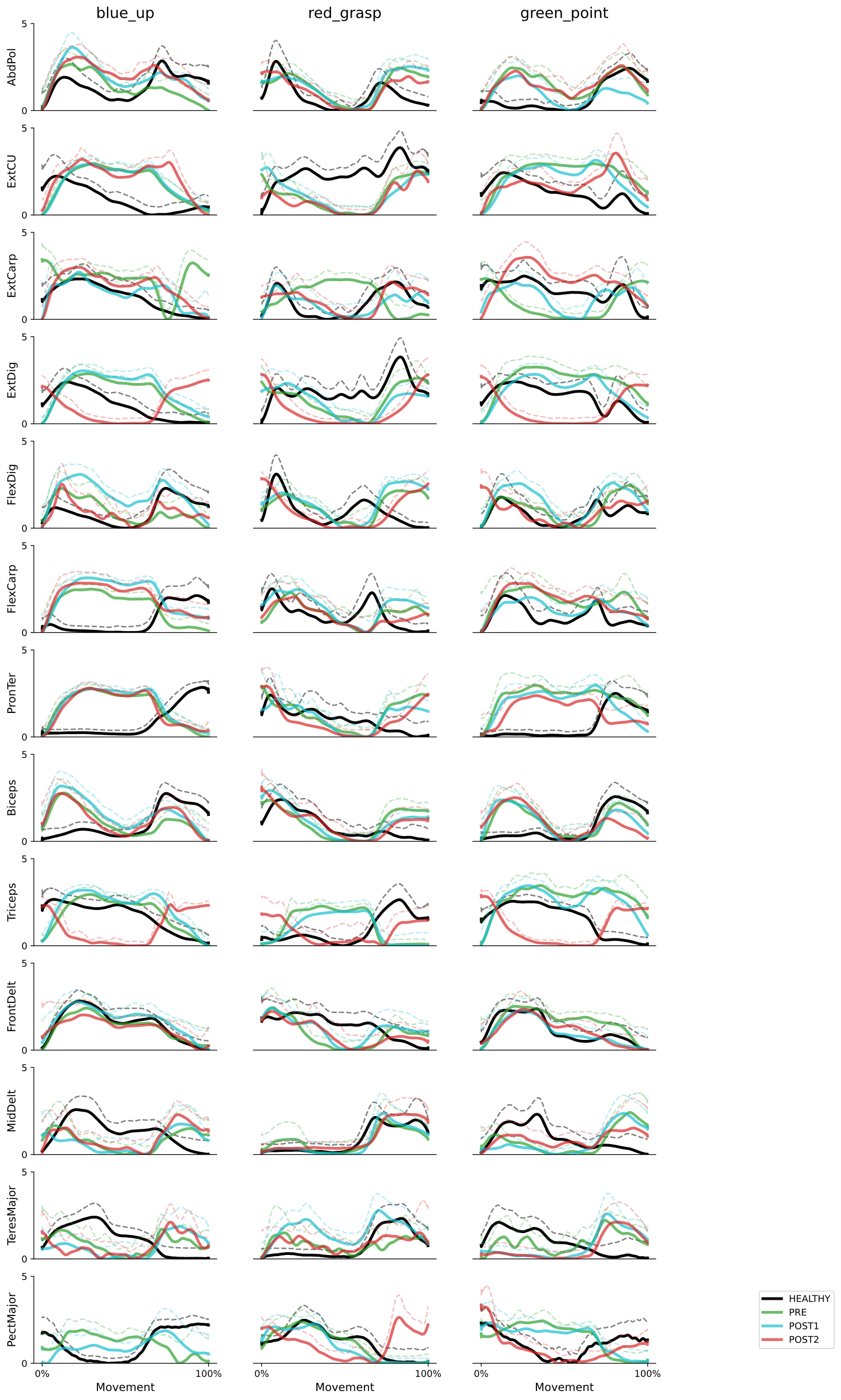


**Supplementary Figure 1.** Task-specific (blue_up, red_grasp, green_point) averaged EMG profiles in healthy (black) and paretic assessments: pre (green), Post1 (blue) and Post2 (red).

## Supplementary Tables

Supplementary Table 1: Classification of Feedback Form Questions into the Different Categories Analyzed. FF*i* refers to the Feedback Form *i*. Q*i* refers to the question number *i* within each Feedback Form. The (-) sign indicates that the question was inversed for the analysis.

| **1- Exo functioning** | **2- Exo ergonomy** | **3-Exo operation** | **4- Exo feedback** | **5- Experimental protocol** | **6- Experimenter** | **7- Performance perception & expectations of improvement** |
| --- | --- | --- | --- | --- | --- | --- |
| FF1-Q5 | FF3-Q3 | - FF1-Q9 | FF1-Q7 | FF1- Q1 | FF2- Q1 | FF1- Q2 |
| FF1-Q6 | FF3-Q4 | FF1- Q10 | FF1- Q8 | - FF3- Q5 | FF2- Q2 | FF1- Q3 |
| FF3-Q1 |  | FF1- Q12 | FF1- Q11 | - FF3- Q9 | - FF2- Q3 | - FF1- Q4 |
| FF3-Q2 |  | - FF3- Q6 | FF3- Q21 | - FF3- Q10 | FF2- Q4 | FF2- Q12 |
|  |  | - FF3- Q7 | - FF3- Q22 | FF3- Q11 | FF2- Q5 | FF2- Q13 |
|  |  | - FF3- Q8 | FF3- Q23 | FF3- Q12 | FF2- Q6 | FF2- Q14 |
|  |  |  | FF3- Q24 | FF3- Q13 | - FF2- Q7 | FF2- Q15 |
|  |  |  |  | - FF3- Q14 | - FF2- Q8 |  |
|  |  |  |  | - FF3- Q15 | - FF2- Q9 |  |
|  |  |  |  | - FF3- Q16 | FF2- Q10 |  |
|  |  |  |  | - FF3- Q17 | FF2- Q11 |  |
|  |  |  |  | - FF3- Q18 |  |  |
|  |  |  |  | - FF3- Q19 |  |  |
|  |  |  |  | - FF3- Q20 |  |  |
|  |  |  |  | FF3- Q25 |  |  |

Supplementary Table 2: Intensity used for each assessment block represented as percentage (%) of the maximum stimulator output.

| **Patient** | **Pre1** | **Post1** | **Post2** |
| --- | --- | --- | --- |
| **1** | 50 % (discomfort) | 50 % (discomfort) | 45 % |
| **5** | 90 % | 90 % | 88 % |
| **6** | 84 % | 73 % | 76 % |
